# Effects of *spoIIE* and *rsfA* Knockout on Spore Formation, Cell Growth, 2,3-Butanediol Synthesis and Heterologous Protein Expression in *Bacillus licheniformis*

**DOI:** 10.3390/microorganisms14040754

**Published:** 2026-03-27

**Authors:** Jinlian Li, Fengxu Xiao, Liang Zhang, Guiyang Shi, Youran Li

**Affiliations:** 1 Key Laboratory of Carbohydrate Chemistry and Biotechnology of Ministry of Education, School of Biotechnology, Jiangnan University, Wuxi 214122, China; 6230201018@stu.jiangnan.edu.cn (J.L.); 8202306022@jiangnan.edu.cn (F.X.); zhangl@jiangnan.edu.cn (L.Z.); gyshi@jiangnan.edu.cn (G.S.); 2 National Engineering Research Center of Cereal Fermentation and Food Biomanufacturing, Jiangnan University, Wuxi 214122, China

**Keywords:** *Bacillus licheniformis*, spore formation rate, gene editing, HPLC, fluorescence intensity detection

## Abstract

Sporulation represents a complex metabolic reprogramming process in bacteria. In this study, we used CRISPR-Cpf1 to knock out *spoIIE* and *rsfA* in *Bacillus licheniformis*. The Δ*spoIIE* strain completely lost sporulataion capacity, while Δ*rsfA* showed a 25% reduction. Although viable cell counts decreased by 80.7% and 45.7%, respectively, glucose consumption and 2,3-butanediol synthesis remained unchanged, and acetoin synthesis increased by 19% in Δ*spoIIE*. Per-cell metabolic rates were significantly enhanced: glucose uptake increased 2.7–3.4-fold, acetoin synthesis 2.3–4.2-fold, 2,3-butanediol synthesis 1.7-fold, and heterologous protein expression 10–15-fold. These findings demonstrate that blocking sporulation liberates metabolic resources and enhances the specific productivity of vegetative cells, providing a strategy for engineering high-performance *B. licheniformis* cell factories.

## 1. Introduction

Bacterial spores, formed by *Bacillus* and *Clostridium* species under stress, exhibit extreme resistance to heat, radiation, and chemicals [1]. *Bacillus licheniformis* serves as an excellent model for studying sporulation due to its efficient sporulation, industrial relevance, and genetic tractability [2]. This complex developmental process, encompassing stages from initiation to maturation [3,4], significantly redirects cellular resources, impacting the synthesis of metabolites like 2,3-butanediol and industrial enzymes [5,6].

Sporulation is governed by a hierarchical genetic cascade (e.g., *spo0A*, *sigF*, *sigE*) [7,8], regulating hundreds of genes [9,10,11,12,13,14,15,16,17,18]. The master regulator Spo0A represses pathways such as acetoin synthesis [19], while *B. licheniformis* uniquely utilizes the glyoxylate cycle to recycle metabolites for energy balance [20]. Previous studies show that disrupting sporulation genes (e.g., *spo0A*, *spoIIE*, *rsfA*) alters metabolism and enzyme production [21,22,23,24]. However, how sporulation or its absence specifically impacts cell growth, product metabolism profiles, and heterologous protein expression capacity remains poorly understood and often confounded by pleiotropic regulatory effects [25].

Accurate sporulation quantification is essential. While methods like heat-treatment plating and qPCR exist [26,27,28,29], they require optimization for reliability [30,31]. To directly elucidate the link between sporulation and anabolic capacity, we employed a CRISPR-Cpf1 system to disrupt key sporulation genes (*spoIIE*, *rsfA*) in *B. licheniformis.* We established a robust sporulation assay and performed multidimensional phenotyping to elucidate inhibitory effects of sporulation gene deletion on spore formation, characterize associated carbon metabolism alterations, and quantify impacts on heterologous protein expression using eGFP as a reporter.

The research aims of this study are as follows:

(1) To use the CRISPR-Cpf1 system to knock out the sporulation genes *spoIIE* and *rsfA* in *Bacillus licheniformis*, and to establish a reliable method for quantitatively measuring sporulation rates to assess changes in recombinant strains’ spore production, while observing morphological changes in the recombinant strains under a microscope.
(2) To use HPLC to detect and determine differences in metabolite profiles between sporulation-deficient strains and the parental strain, and to analyze the impact of sporulation deficiency on metabolic changes.
(3) To assess fluorescence intensity by expressing the green fluorescent protein eGFP, and analyze differences in heterologous protein expression between sporulation-deficient strains and the parental strain.

Collectively, this work provides novel engineering strategies and technical frameworks for enhancing production capabilities in *B. licheniformis*.

## 2. Materials and Methods

Experimental Material Strains and Plasmids. The bacterial strains *Escherichia coli* JM109 and *Bacillus licheniformis* CICIM B1319, along with the plasmids pJOE8999-Cpf1 and epWBN-p2 Egfp used in this study, were obtained from laboratory stocks. *Escherichia coli* JM109 was employed for the construction of all knockout plasmids in this research. All strains and plasmids used in this study can be found in Table 1.

Enzymes and Main Reagents. The reagents and kits required for this experiment are shown in Table 2.

Media and Reagent Preparation. The media used in this experiment include synthetic media (glucose 30 g/L, urea 10 g/L, K_2_ HPO_4_ 10 g/L, KH_2_ PO_4_ 1.36 g/L, MgSO_4_·H_2_O 2 g/L, (NH_4_)_2_ HPO_4_ 5 g/L, trace elements 2 g/L, sodium glutamate 10 g/L), LB medium 1 (glucose 30 g/L, yeast extract 5 g/L, peptone 10 g/L, sodium chloride 10 g/L), and LB medium 2 (yeast extract 5 g/L, peptone 10 g/L, sodium chloride 10 g/L). For the preparation of solid LB medium, agar powder was typically used at approximately 2%. All media were sterilized at 115 °C for 20 min. Kanamycin was added as needed to a final concentration of 30 μg/mL. The media and reagents used for electroporation of *Bacillus licheniformis* are: Medium I: liquid LB medium supplemented with 0.5 M sorbitol. Recovery medium: liquid LB medium supplemented with 0.5 M sorbitol and 0.38 M mannitol. Buffer BW: 0.5 M sorbitol, 0.5 M mannitol, 10% glycerol.

Experimental Equipment. The equipment used in this study is shown in Table 3.

Primer Design. The primers used in this study are listed in Table 4. Primers were designed using SnapGene v6.0.2 software, and both primer synthesis and DNA sequencing services were provided by Shanghai Sangon Biological Engineering Co., Ltd. (Shanghai, China).

Experimental Methods. CRISPR-Cpf1 Target Sequence Design. The design of accurate and efficient crRNA plays a crucial role in the CRISPR-Cpf1 mediated gene knockout system. The crRNA guides Cpf1 to specific sites within the target gene, where its nuclease activity cleaves DNA to form double-strand breaks (DSBs). Cells repair DSBs through error-prone non-homologous end joining (NHEJ), resulting in insertion or deletion mutations that disrupt the gene coding frame or functional domains, thereby achieving gene knockout. To minimize off-target binding and topological effects, all experimental targets were designed within the exon regions of sporulation genes, avoiding promoter or conserved functional domain regions, while ensuring the presence of Cpf1-compatible protospacer adjacent motifs (PAMs) near the target sites. Specific designs were performed using CHOPCHOP (CHOPCHOP uib.no) and SnapGene software. All target sequences involved in this study are shown in Table 5.

CrRNA Expression Cassette Construction. Construction of *spoIIE* and *rsfA* Knockout Plasmids. Using the construction of the *spoIIE* knockout plasmid as an example, the linearized vector pJOE8999 was first obtained through SmaI and BamHI digestion. Following enzymatic treatment, the DNA products were purified through gel electrophoresis recovery to obtain high-purity target fragments, and their concentrations were determined. Using primers *spoIIE*-Left-F/R and *spoIIE*-Right-F/R with *Bacillus licheniformis* genomic DNA as template, fragments 1 and 2 were amplified by PCR. Subsequently, using primers *spoIIE*-Left-F and *spoIIE*-Right-R with the aforementioned fragments 1 and 2 as templates, the crRNA expression cassette sequence containing homologous arms was amplified through overlap extension PCR. The purified target fragment was inserted into the restriction enzyme cleavage sites of linearized vector pJOE8999 via homologous recombination and transformed into *E. coli* JM109 competent cells. To verify transformation results, 10–20 transformants were randomly selected for colony PCR identification. Furthermore, plasmid DNA was extracted from these transformants and their concentrations were determined. Using primers *spoIIE*-crRNA-F/R with the extracted plasmid DNA as template, the target gene sequence was amplified by PCR. After fragment purification, DpnI enzyme was used to eliminate the original plasmid, and the digested DNA was transformed again into *E. coli* JM109 competent cells. Ten to twenty transformants were randomly selected for colony PCR identification, and Sanger sequencing was employed to confirm the sequence accuracy of the constructed recombinant plasmid. The final knockout plasmid targeting sporulation genes, pJOE8999 Ppro-cas12a-*spoIIE* (pE), was obtained. The *rsfA* knockout plasmid (pA) was constructed following the same procedure. Throughout this process, molecular biology operations including *B. licheniformis* genomic DNA extraction, PCR product purification, DNA gel recovery, and plasmid extraction were performed strictly according to the instructions of the corresponding reagent kits.

Molecular Experimental System Preparation and Reaction Conditions. PCR amplification conditions: 95 °C pre-denaturation 5 min/30 s, 95 °C denaturation 30 s/15 s, 55 °C annealing 30 s/15 s, 72 °C extension (calculated according to the corresponding enzyme amplification efficiency), 19–35 cycles, final extension 5 min. Double enzyme digestion reaction system and conditions: Enzyme I 2 μL, Enzyme II 2 μL, 10× Buffer 5 μL, target fragment and ddH_2_ O totaling 41 μL. 37 °C, 2 h. Homologous recombination ligation system and conditions: 5× all-in-one homologous recombination enzyme 1 μL, 5× Buffer 2 μL, fragment mass (ng) = fragment length (bp) × 0.04, vector mass (ng) = vector length (bp) × 0.02. 37 °C, 30 min.

Construction of Sporulation Knockout Strains. Preparation of *B. licheniformis* Competent Cells. Following the method of Zhao Xinxin et al. [32], strain B1319 was streaked on antibiotic-free LB plates the night before and cultured for 12–16 h. A single colony was picked and inoculated into 15 mL LB liquid medium, followed by overnight cultivation at 37 °C and 250 rpm for 16 h. One milliliter was inoculated into medium I and cultured at 37 °C and 250 rpm for 3–4 h, after which cells were collected by centrifugation at 8000 rpm for 5 min. Each tube of cells was washed with 20 mL BW solution, and this washing procedure was repeated 2–3 times. After the final wash, 750 μL BW was added to the samples, which were then distributed into individual 1.5 mL centrifuge tubes at 80 μL per tube. After aliquoting, samples were stored at −70 °C in an ultra-low temperature freezer for subsequent experiments.

Construction of Knockout Strains Δ*spoIIE* and Δ*rsfA*. The constructed and validated pE and pA plasmids were first electrotransformed into B1319 competent cells according to the method of Xiao et al. [33], and recombinant strains containing pE and pA were obtained by plating on kanamycin-resistant LB plates. Transformants were then selected and verified by colony PCR using Δ*spoIIE*-YZ-F/R and Δ*rsfA*-YZ-F/R primers, respectively. The verified colonies were designated as BLpE and BLpA, and gene knockout strains with plasmid loss were obtained after antibiotic elimination [34]. Knockout efficiency was evaluated by randomly selecting transformants from each transformation, and knockout strains were identified through PCR, gel electrophoresis, and Sanger sequencing [35]. The obtained knockout strains Δ*spoIIE* and Δ*rsfA* were stored at −70 °C, and competent cells were prepared for future use.

Phase-contrast microscope. Phase contrast microscopy was performed using the XSP 13C-LP microscope equipped with a Ph3 DL 100x/1.25 oil immersion phase contrast objective. After appropriate dilution, the 48 h cultured bacterial suspension is placed onto the center of a slide and covered with a coverslip. Then, the vegetative cells and spore morphological differences are observed using an oil immersion lens while the microscope settings are adjusted.

Quantitative Detection Method for Spore Formation Rate. Heat treatment time determination: Spore formation rates were assessed at 6 h and 12 h using three common heat treatment durations (10 min, 15 min, and 30 min). Detection conditions included cultivation in LB2 medium at 37 °C with 220 rpm agitation, heat treatment at 80 °C, and an OD_600_ of approximately 2. Medium selection: Using the optimized heat treatment duration determined above, spore formation rates of wild-type *Bacillus licheniformis* were evaluated over 0–96 h in synthetic medium, LB1, and LB2. Detection conditions were 37 °C cultivation with 220 rpm agitation, heat treatment at 80 °C for 10 min in a water bath. Final detection protocol: Knockout strains were cultivated in LB2 medium at 37 °C with 220 rpm agitation for 0–96 h, with sampling every 12 h/24 h. Heat treatment was performed at 80 °C for 10 min in a water bath at an OD_600_ of approximately 2. The number of colonies plated after heat treatment, multiplied by the dilution factor, was recorded as the spore count, while the number of colonies plated without treatment, multiplied by the dilution factor, was recorded as the total viable cell count. Spore formation rate (%) = spore count/total colony count × 100%.

HPLC Metabolite Detection. *Bacillus licheniformis* B1319, Δ*spoIIE*, and Δ*rsfA* strains were streaked and activated, then a loopful of cells was inoculated into 15 mL LB liquid medium for overnight cultivation. The following day, overnight cultures were transferredto synthetic medium at a 3% (*v*/*v*) inoculation ratio and cultivated at 37 °C with 220 rpm orbital shaking. Samples were collected for OD_600_ measurement and post-fermentation HPLC detection of glucose, acetoin, and 2,3-BD. Detection procedure: Following the method of Sanson et al. [36], 1 mL samples were centrifuged at 12,000 rpm for 5 min, the pellet was discarded, and the supernatant was retained. An equal volume of 10% trichloroacetic acid was added (1:1 ratio), and the mixture was incubated at 4 °C for at least 4 h for precipitation. The mixture was then centrifuged at 12,000 rpm for 30 min to achieve complete solid–liquid phase separation. Chromatographic conditions: RI detector, chromatographic column (8 × 300 mm, Dikma CarboPac Ca^2+^ 6 μm), mobile phase of ultrapure water, flow rate of 0.8 mL/min, column temperature of 80 °C, and run time of 20 min. Quantification method: External standard curve method.

Fluorescence Intensity Detection. The fluorescent plasmid epWBN-P2-eGFP was electroporated into competent B1319, Δ*spoIIE*, and Δ*rsfA* cells, followed by verification using primers epWBN-eGFP-F/R. Correct transformants were selected, cultured in shake flasks, and inoculated into synthetic medium for cultivation at 37 °C with 220 rpm agitation for 0–72 h. Samples were collected for OD_600_ and fluorescence intensity detection. Excitation wavelength 480 nm, emission wavelength 520 nm, with gain value (Gain = 60) and absorbance (600 nm) measurements.

Statistical Analysis. All experiments were independently repeated three times, and the average value was taken as the final result. The differences between two sets of data were analyzed using a 2-tailed Student’s *t*-test, while the differences between multiple sets of data were compared using one-way ANOVA and Tukey’s test. “*” and “***” were used to indicate the significance of *p* < 0.05 and *p* < 0.001, respectively.

## 3. Results

### 3.1. Screening and Identification of Key Sporulation Genes

Through literature review, sporulation-related genes in *Bacillus licheniformis* were identified as shown in Table 6.

Literature review reveals that sporulation is a complex multigene regulatory process involving genes that either influence master regulatory factors or directly participate in specific stages of spore formation. Among these, the *spoIIE* gene has been extensively studied. This gene is primarily expressed during stage II of sporulation and is ubiquitously present in sporulating bacteria, regulating the formation of asymmetric membranes during stage II sporulation and affecting the separation of mother cells from prespore compartments. SpoIIE functions as a phosphatase, participating in the activation of the prespore-specific transcription factor σ^F^. Studies in numerous *Bacillus* species have demonstrated that genetic modification of *spoIIE* affects spore formation rates, growth enzyme activity, and the synthesis of metabolites such as 2,3-butanedio [15,22,37,38]. In contrast, *rsfA* has received limited research attention. However, as shown in Table 6, this gene operates at a different stage of sporulation compared to *spoIIE*. The *rsfA* gene is primarily expressed in prespores under σ^F^ factor regulation and indirectly influences spore formation. Wu et al. confirmed that this gene fine-tunes gene expression in spores, with its deletion reducing sporulation rates in *B. subtilis* 168 by approximately 30% [24]. However, no studies have validated whether this gene affects spore formation rates, protein expression, and major metabolic changes in *Bacillus licheniformis*. Therefore, selecting this gene to investigate the relationship between sporulation and bacterial growth metabolism offers both representativeness and significant research value. The selection of *spoIIE* and *rsfA* genes—which affect different stages of sporulation and have varying degrees of research foundation and exploration depth—enables a multifaceted investigation of the correlations between sporulation and growth-metabolic phenotypes in *B. licheniformis*. This approach holds important implications for the industrial development and design of *B. licheniformis* strains.

### 3.2. Construction of Sporulation-Deficient Knockout Strains ΔspoIIE and ΔrsfA Using the CRISPR-Cpf1 System

**Figure 1:**
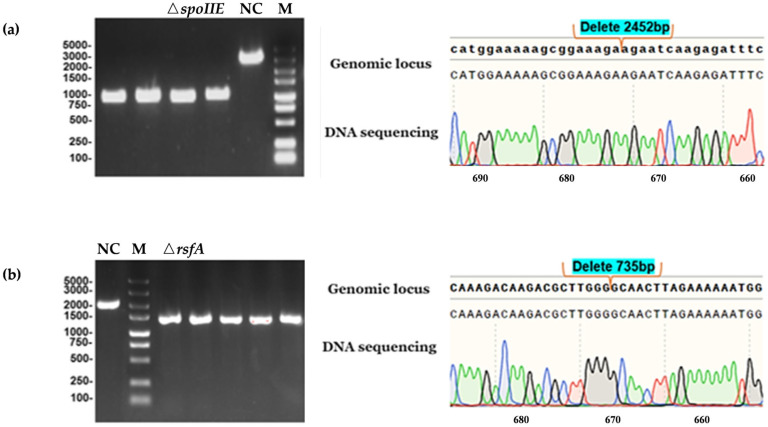
Knockout strain colony PCR and sequencing results. M-DNA Marker 5000 bp; DNA sequence chromatogram of *spoIIE* and *rsfA*. The four nucleotides are color-coded as follows: C (cytosine) in blue, A (adenine) in green, G (guanine) in black, and T (thymine) in red. (**a**) Verification of the Δ*spoIIE* mutant colony by PCR and gel electrophoresis, and comparison with sequencing results. A total of 2452 bp was deleted, which is consistent with the expected fragment size; (**b**) Verification of the Δ*rsfA* mutant colony by PCR and gel electrophoresis, and comparison with sequencing results. A total of 735 bp was deleted, which is consistent with the expected fragment size.

Recombinant plasmids were successfully constructed in *B. licheniformis* using the CRISPR-Cpf1 system, with a plasmid size of 8424 bp. Double digestion of the plasmid with BamHI and SmaI should theoretically yield fragments of 7420 bp and 1004 bp. Electroporation of the recombinant plasmids into *B. licheniformis* B1319 generated recombinant strains BLpE and BLpA. The *spoIIE* and *rsfA* genes in *B. licheniformis* B1319 are 2490 bp and 786 bp in size, respectively. Following gene knockout and multiple passages of screening, colony PCR using Δ*spoIIE*-YZ-F/R and Δ*rsfA*-YZ-F/R primers should produce single bands of theoretical sizes 1130 bp and 1416 bp, respectively, representing sequence deletions of 2452 bp and 735 bp compared to the control strain. After plasmid loss and subsequent PCR and sequencing, as shown in Figure 1, bands consistent with the theoretical sizes were observed, confirming successful construction of the knockout strains.

Experimental results revealed variable gene knockout efficiencies when using the CRISPR-Cpf1 system for gene editing, with the sporulation gene *spoIIE* achieving 100% knockout efficiency while *rsfA* achieved 40% efficiency. The editing efficiency of the CRISPR-Cpf1 system is closely associated with promoter type, target site design, and competent cell status. Liu et al. [39] explicitly demonstrated during CRISPR-Cpf1 system construction in *Bacillus licheniformis* that rigorous selection of high-efficiency expression elements for conditional Cpf1 protein expression is critical for successful target strain construction. The Cpf1 protein and crRNA in the CRISPR-Cpf1 system require optimal expression levels; insufficient expression leads to poor cleavage efficiency and editing failure, while sustained high expression may cause cellular toxicity and increase off-target risks [40]. Therefore, selecting promoters with high host cell compatibility that provide appropriate expression dynamics is crucial. For instance, in *Corynebacterium glutamicum*, researchers found it necessary to optimize induction systems to coordinate the timing of Cpf1 expression and recombination events [41]. Hao et al. [42] conducted knockout validation in *Bacillus subtilis* and concluded that CRISPR-Cpf1 outperformed CRISPR-Cas9 strategies in single-gene knockouts, achieving editing efficiencies up to 100%. When constructing sporulation gene knockout strains in *B. licheniformis* using the CRISPR-Cpf1 system, several key strategies were identified for improving editing efficiency: First, designing highly specific targets that comply with Cpf1 PAM sequence requirements (TTTV) while avoiding potential off-targets, with reference to reliable crRNA sequences during design; Second, optimizing promoter selection, such as using maltose-inducible promoters or the proline-constitutive strong promoter described in this study, testing different induction timepoints and inducer concentrations to identify optimal expression windows that balance editing efficiency and cell viability while ensuring high-level Cpf1 and crRNA expression; Third, utilizing highly efficient competent cells to achieve high transformation efficiency for electroporation. Additionally, plasmid curing after gene knockout typically requires passages [43], with appropriate temperature elevation during passaging facilitating plasmid loss.

### 3.3. Cell Morphology Before and After Sporulation Gene Knockout

**Figure 2:**
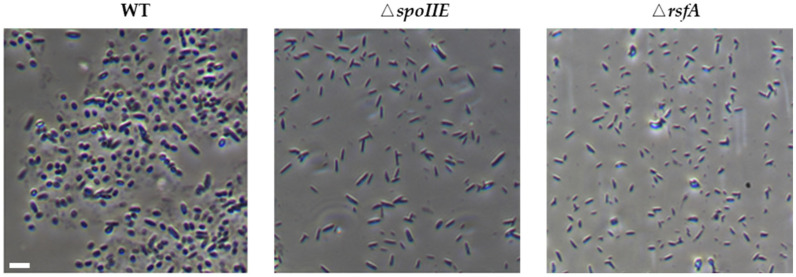
The cell morphology of the strains was observed using a phase-contrast microscope. Scale bar = 2 µm. Images were captured using a 100x oil immersion phase contrast objective (Ph3 DL, 100x/1.25). Under the same culture conditions, the differences in cell morphology between the wild-type (WT) strain and the two sporulation-deficient mutants (Δ*spoIIE* and Δ*rsfA*) were observed.

The cell morphology of the strains was obtained using phase-contrast microscopy, as shown in Figure 2. Under the same culture conditions, the wild-type (WT) strain and two sporulation-deficient mutants (Δ*spoIIE* and Δ*rsfA*) exhibited significant morphological differences. The wild-type strain exhibits a typical and regular short rod or rod-shaped morphology. In cells undergoing the sporulation process, the formation of asymmetric septa and highly refractive oval endospores can be clearly observed, indicating that the sporulation process is proceeding normally. The Δ*spoIIE* mutant, after the deletion of the key regulatory gene *spoIIE* responsible for asymmetric division and spore development, is unable to form mature spores. The cell morphology changes significantly, with most cells exhibiting markedly elongated rod-shaped or filamentous structures, and a lack of normal septa internally, clearly indicating that spore formation is blocked at an early stage. The Δ*rsfA* mutant strain similarly leads to a phenotype with defective spore formation. The cells of this mutant have irregular shapes; although some still maintain a short rod-like form, abnormalities such as twisting, swelling, or narrowing are observed. Additionally, incomplete septum structures can be seen inside the cells, and they completely lack highly refractive mature spores, indicating defects in spore maturation or cortex formation.

Compared with the wild-type strain, both spore-deficient mutants exhibited morphological differences. However, the Δ*spoIIE* mutant was characterized by significantly elongated cells and almost no spore formation, whereas the Δ*rsfA* mutant mainly displayed irregular morphology due to unstable cell membranes, but still produced spores. These morphological observations support the roles of SpoIIE and RsfA at different stages of sporulation and help us understand the genetic determinants essential for bacterial spore formation.

### 3.4. Establishment of Sporulation Rate Detection Method in Bacillus licheniformis

**Figure 3:**
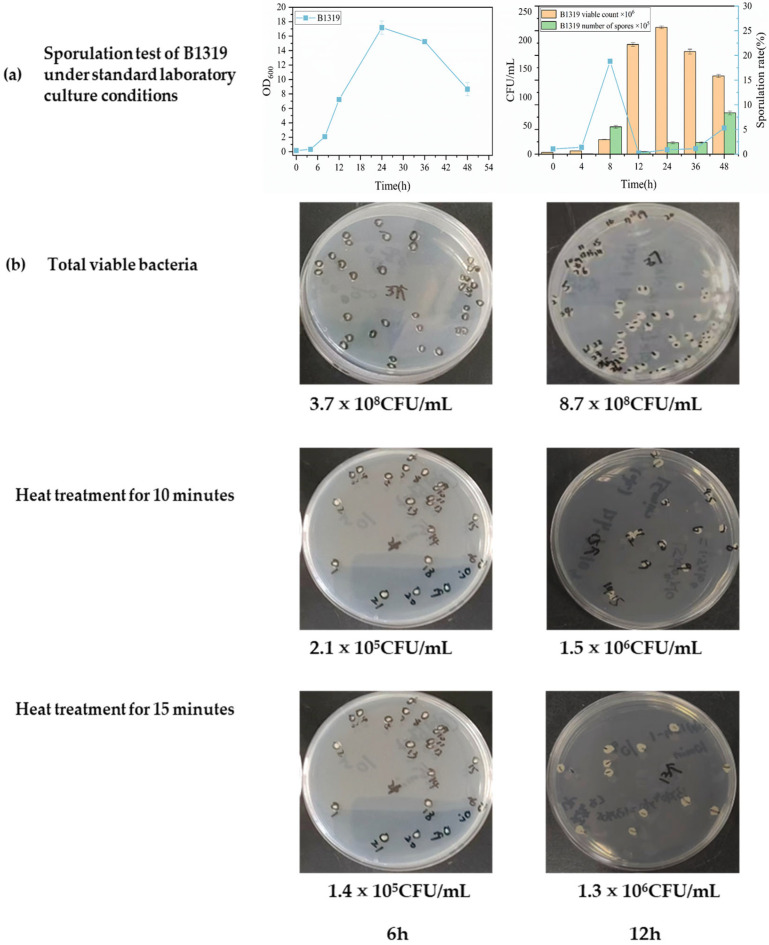
Determination of key detection parameters for the growth curve and spore formation rate of *Bacillus licheniformis* Wild-Type Strain B1319. (**a**) Synthetic medium subjected to heat treatment at 80 °C for 30 min; (**b**) Plate colony images after 10 min and 15 min of heat treatment.

**Figure 4:**
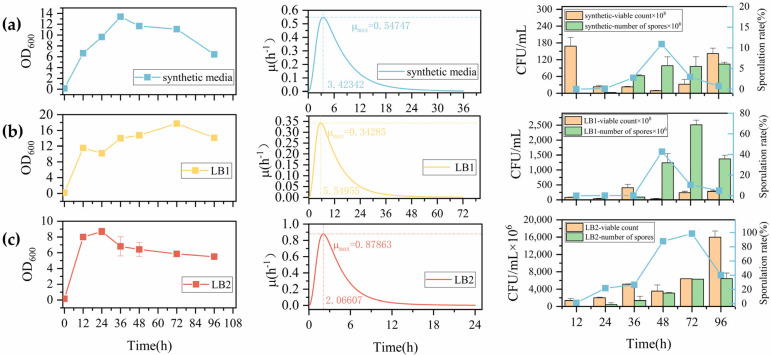
Heat treatment for 10 min under different medium conditions. In the rightmost column of the graphs, there are two Y-axes. To distinguish the meanings represented by the different Y-axes, the blue line represents the spore formation rate, corresponding to the blue line in the graph. (**a**) Growth curve, specific growth rate, and spore formation rate detection under synthetic medium conditions; (**b**) Growth curve, specific growth rate, and spore formation rate detection under LB medium conditions with 30 g/L glucose; (**c**) Growth curve, specific growth rate, and spore formation rate detection under basic LB medium conditions.

Key parameters including fermentation medium type and heat treatment duration were determined through multiple experiments with wild-type *B. licheniformis*, followed by detection of sporulation rates in recombinant strains using the established sporulation rate detection method. Experimental results are shown in Figure 3 and Figure 4.

Wild-type *B. licheniformis* OD_600_ and sporulation rates were initially assessed in synthetic medium over 0–48 h under conditions of 80 °C heat treatment for 30 min with bacterial suspension heat treatment concentration at OD_600_ = 2. As shown in Figure 3a, bacterial suspension OD_600_ peaked at 24 h, reaching 17.175; under unchanged heat treatment temperature and bacterial concentration conditions, maximum viable cell count was 2.225 × 10^8^ CFU/mL with spore count of 2 × 10^6^ CFU/mL, yielding a sporulation rate of 0.9%. These results indicated that while the standard laboratory medium supports sporulation, it is suboptimal for bacterial accumulation and sporulation rate detection. Additionally, excessive heat treatment duration resulted in killing of some heat-sensitive spores, leading to artificially low sporulation rates. These experimental results are consistent with the spore heat resistance results reported by Sohyeon Kim and others [44], so it is necessary to adjust the type of culture medium and shorten the heat treatment time in subsequent spore formation rate tests. Initial experiments confirmed that *Bacillus licheniformis* begins accumulating biomass during the logarithmic phase, when cells in the culture are predominantly in the vegetative state with relatively few spores present. During the late growth phase, spores form abundantly and increase steadily. To determine the optimal heat treatment duration for detecting spore formation rates, samples were collected at 6 h and 12 h during the logarithmic phase under identical conditions to the initial experiment, followed by heat treatment of the cell suspension for 10 min and 15 min. Plate counting results were used to compare the effectiveness of different heat treatment durations in inactivating vegetative cells of *B. licheniformis*. As shown in Figure 3b, biomass accumulation increased from 6 to 12 h, and both 10 min and 15 min heat treatments achieved greater than 95% mortality of vegetative cells in the suspension. However, the 10 min heat treatment resulted in significantly higher spore counts in the suspension, leading to the selection of 10 min as the optimal heat treatment duration. Combining results from both experiments, bacterial growth and spore formation rates of *B. licheniformis* were evaluated in LB medium 1, LB medium 2, and synthetic medium under conditions of 80 °C heat treatment for 10 min at a cell suspension concentration of OD_600_ = 2, to determine the most suitable medium type. As shown in Figure 4, in synthetic medium, the cell suspension OD_600_ reached its maximum of 13.4 at 36 h, while spore formation rate peaked at 48 h at 10.9%, with viable cell counts of 9.05 × 10^8^ CFU/mL and spore counts of 9.865 × 10^7^ CFU/mL. Notably, reducing heat treatment duration under identical conditions significantly enhanced spore formation rates; however, synthetic medium remained unsuitable for spore formation, maintaining relatively low spore formation rates. In glucose-containing LB1 medium, the cell suspension OD_600_ reached its maximum of 17.74 at 72 h, but the specific growth rate peaked at 0.34 h^−1^ only at 0.55 h, representing a decreased specific growth rate compared to the other two culture conditions and a delay of over 2 h in reaching maximum specific growth rate. Spore formation rate peaked at 48 h at 42.7%, with viable cell counts of 2.9 × 10^9^ CFU/mL and spore counts of 1.237 × 10^9^ CFU/mL. The experiment demonstrated that under nutrient-rich conditions, viable cell counts increased; however, since spores typically form under nutrient-limited conditions, the lag phase for spore formation under nutrient-rich conditions extended to 24 h, with formation rates significantly lower than those observed in glucose-free LB2 medium under equivalent conditions. In glucose-free LB2 medium, the cell suspension OD_600_ reached its maximum of 8.7 at 24 h, with specific growth rate peaking at approximately 0.88 h^−1^ around 2 h. This medium achieved maximum spore formation rates of approximately 99% at 72 h, with viable cell counts of 6.4 × 10^9^ CFU/mL and spore counts of 6.313 × 10^9^ CFU/mL, making it most suitable for spore formation rate detection. Comparison of growth and sporulation rates of wild-type *Bacillus licheniformis* across three culture media revealed two key phenomena. First, OD_600_ measurements exhibit limitations in monitoring spore-forming bacterial growth, with viable cell counts providing a more accurate reflection of *B. licheniformis* growth dynamics. The growth of *B. licheniformis* is significantly influenced by sporulation processes. Unlike non-spore-forming strains such as *Escherichia coli*, spore-forming bacteria exhibit extended growth phases, and cultivation time critically affects sporulation rate detection. Analysis of viable cell and spore count variations demonstrates that spore formation prolongs the bacterial growth cycle, with substantial colony formation persisting even after nutrient depletion. During the lag phase through early death phase, viable cell count changes remain synchronized with OD_600_ values. However, following the death phase, extensive spore formation in *B. licheniformis* extends the growth period and alters cellular morphology. At this stage, OD_600_ decreases due to reduced light scattering efficiency while viable cell counts increase, resulting in CFU measurements showing an upward trend during 48–96 h that is asynchronous with OD_600_ values. Research indicates that OD_600_ measurements reflect the turbidity of total bacterial mass, including vegetative cells, spores, and dead cells, whereas viable cell counts (CFU) represent reproductively capable living cells. During the late stationary and death phases of spore-forming strains, culture turbidity decreases or remains constant due to spore formation and vegetative cell autolysis, while spore numbers may be high and stable. Throughout the process from spore formation initiation to peak sporulation rates, OD_600_ and sporulation rate trends are not synchronized, preventing OD_600_ from providing a complete description of spore-forming bacterial growth [43]. Additionally, cultivation time significantly impacts spore formation. Before 36 h, spore numbers remain relatively low, indicating suboptimal conversion rates. However, extending cultivation time to 48–72 h results in significant spore number increases, demonstrating that spore formation and maturation reach relatively stable states during this period. Further extension to 96 h reveals declining spore numbers and conversion rates, reflecting the adverse effects of bacterial aging on sporulation. Wang et al. [45]. found that *Bacillus subtilis* sporulation rates peaked at 67.7% on the third day of cultivation, subsequently showing a declining trend. These findings align with our experimental results (Figure 4), confirming that sporulation rate detection requires appropriate extension of bacterial cultivation time. Second, compared to synthetic media, sugar-free LB2 medium with peptone as the primary nitrogen source proved significantly more suitable for sporulation rate detection. Under identical time and nutrient-rich conditions, total viable cell counts increased 2–10-fold, though spore formation showed delayed onset. Culture medium composition significantly influences bacterial spore yield, with carbon source concentration serving as a critical factor affecting spore production. Wang et al. [45] investigated carbon/nitrogen source concentration optimization for high spore production in *B. licheniformis* BF-002, finding that glucose as a carbon source resulted in faster growth rates and higher spore yields compared to starch. Khardziani et al. examined key factors affecting *B. subtilis* sporulation and found peak spore numbers of 2.3 × 10^9^ CFU/mL at glucose concentrations of 2 g/L [46]. However, further increases in carbon source concentration led to declining spore yields. Different nitrogen sources also significantly influence spore formation. Comparative studies of various inorganic salts and organic compounds as nitrogen sources revealed substantial differences in spore production under different nitrogen conditions. KNO_3_ emerged as the optimal inorganic nitrogen source, increasing spore numbers 2-fold compared to controls. In contrast, all organic nitrogen sources favored spore production, with peptone as the nitrogen source achieving the highest spore accumulation at 6.6 × 10^10^ CFU/mL. Sporulation represents a response to environmental stresses including nutrient limitation (particularly carbon and nitrogen starvation) and elevated temperatures, though carbon/nitrogen source concentration ratios and high-temperature exposure duration significantly influence spore formation. Therefore, the initially used standard laboratory synthetic medium produced spores under conditions of 30 g/L glucose concentration and 30 min high-temperature water bath treatment, but with extremely low sporulation rates. After adjusting the water bath time to 10 min, the synthetic medium achieved an 11% sporulation rate. Among media containing 30 g/L glucose (LB1) and LB2 basal medium, LB2 demonstrated superior sporulation potential, achieving approximately 99% sporulation rates within 72 h. This occurs because LB1 contains excessive carbon source concentrations, depriving cells of the stress signals necessary for spore formation initiation. LB2 provides a carbon-limited environment while supplying peptone as an optimal nitrogen source for spore formation. In summary, the optimal conditions for spore formation rate detection were established as follows: LB2 basal medium, 80 °C water bath for 10 min, and bacterial culture at 37 °C with 220 rpm shaking for 96 h.

### 3.5. Growth, Metabolism, and Spore Formation Rate Analysis of Knockout Strains

**Figure 5:**
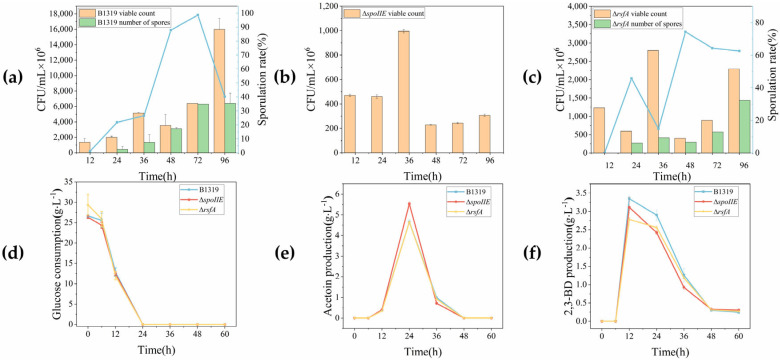
Knockout strain spore formation rate and metabolic analysis. In (**a**,**c**), to distinguish the meanings represented by different Y-axes, the blue line represents the spore formation rate, corresponding to the blue line in the figure. (**a**) 0–96 h Detection of growth and spore formation rate of wild-type strain B1319; (**b**) 0–96 h Detection of growth and spore formation rate of Δ*spoIIE* strain; (**c**) 0–96 h Detection of growth and spore formation rate of Δ*rsfA* strain; (**d**) Glucose consumption curves of B1319, Δ*spoIIE*, and Δ*rsfA* strains from 0 to 60 h; (**e**) Acetoin production of B1319, Δ*spoIIE*, and Δ*rsfA* strains from 0 to 60 h; (**f**) 2,3-BD production of B1319, Δ*spoIIE*, and Δ*rsfA* strains from 0 to 60 h.

Based on the established quantitative detection method for spore formation rate, we evaluated changes in spore formation capacity in the knockout strains. To elucidate growth differences and carbon flux distribution during cultivation, strains were cultured in defined synthetic medium at 37 °C with 220 rpm shaking for 60 h, and major metabolite production was analyzed by HPLC. The spore formation rates and metabolite profiles of the knockout strains are presented in Figure 5.

**Figure 6:**
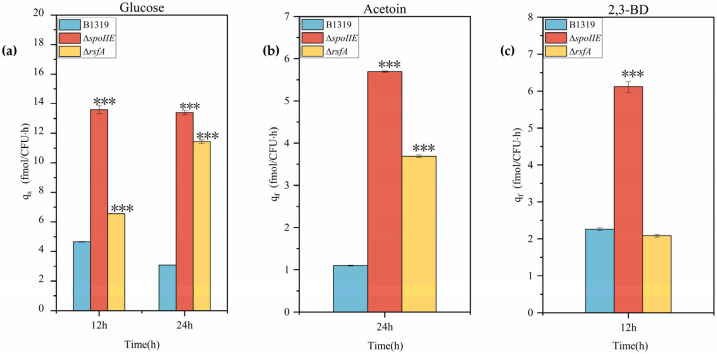
Comparison of the metabolic synthesis capacity per cell of sporulation-deficient strains. (**a**) Specific glucose consumption rate per CFU of the sporulation-deficient strain at 12 and 24 h; (**b**) Specific acetoin biosynthesis rate per CFU of the sporulation-deficient strain at 24 h; (**c**) Specific 2,3-BD biosynthesis rate per CFU of the sporulation-deficient strain at 12 h. All experiments were independently repeated three times, and the average value was taken as the final result. The differences between two sets of data were analyzed using a 2-tailed Student’s *t*-test, while the differences between multiple sets of data were compared using one-way ANOVA and Tukey’s test. “***” is used to indicate a significance of *p* < 0.001.Wild-type strain B1319 and sporulation-deficient strains Δ*spoIIE* and Δ*rsfA* exhibited specific glucose consumption rates per cell of 4.65, 13.61, and 6.55 fmol/(CFU·h), respectively, at 12 h, with corresponding 2,3-BD production rates of 2.26, 6.12, and 2.09 fmol/(CFU·h). At 24 h, the specific glucose consumption rates were 3.09, 13.4, and 11.43 fmol/(CFU·h), respectively, while acetoin production rates reached 1.1, 5.7, and 3.69 fmol/(CFU·h). These results demonstrate that knockout of sporulation genes significantly enhanced the metabolic and biosynthetic capacity per cell. When sporulation is blocked, cellular resources and energy utilization may be redirected toward more efficient metabolic pathways, enabling the cells to achieve several-fold increases in per-cell metabolic capacity despite growth inhibition.

Analysis of spore formation rates revealed that both knockout strains Δ*spoIIE* and Δ*rsfA* exhibited impaired sporulation. The wild-type strain achieved maximum spore formation rate of approximately 99% at 72 h, with viable cell count of 6.4 × 10^9^ CFU/mL and spore count of 6.31 × 10^9^ CFU/mL. In contrast, the Δ*spoIIE* strain showed complete sporulation deficiency (0% spore formation rate), reaching maximum viable cell count of 9.95 × 10^8^ CFU/mL at 36 h. The Δ*rsfA* strain achieved maximum spore formation rate of approximately 74% at 48 h, with viable cell count of 4 × 10^8^ CFU/mL and spore count of 2.97 × 10^8^ CFU/mL. These results demonstrate that knockout of sporulation genes significantly reduced spore formation rates and substantially decreased both total viable cell numbers and spore counts, confirming that both *spoIIE* and *rsfA* genes are critical for spore formation in *B. licheniformis*. Furthermore, sporulation-deficient strains exhibited distinct growth patterns compared to the control strain, with more pronounced growth differences observed for genes with greater importance in spore formation. For instance, at 36 h, viable cell counts decreased by 80.7% for the Δ*spoIIE* strain and 45.7% for the Δ*rsfA* strain. Previous studies by Li et al. demonstrated that knockout of the sporulation gene *spo0A* in *Bacillus subtilis* 168 completely blocked spore formation and reduced cell density by 31.0% [47]. Similarly, Zhang et al. reported that knockout of the *spoIIE* gene in *Bacillus clausii*, compared with the original strain, the sporulation rate of *B.clausii* QL-1Δ*spoIIE* decreased to 0.48% at 28 h and the biomass increased by 20% at 20 h [48]. These findings collectively confirm that impaired spore formation inhibits bacterial growth, with more critical sporulation genes exerting more pronounced inhibitory effects. Despite growth inhibition, the sporulation-deficient strains maintained metabolic capacity and biosynthetic capability, with substantially enhanced per-cell metabolic activity. HPLC analysis revealed similar glucose consumption patterns between sporulation-deficient and control strains, but differences in acetoin and 2,3-BD synthesis. All strains completely consumed 30 g/L glucose within 24 h. Acetoin production peaked at 24 h, with yields of 4.66 g/L, 5.53 g/L, and 4.64 g/L for wild-type, Δ*spoIIE*, and Δ*rsfA* strains, respectively. Maximum 2,3-BD production occurred at 12 h, with yields of 3.35 g/L, 3.11 g/L, and 2.78 g/L for wild-type, Δ*spoIIE*, and Δ*rsfA* strains, respectively. While glucose consumption and major metabolite production appeared similar between sporulation-deficient and control strains, considering the observed reduction in viable cell counts due to impaired sporulation and the limitation of OD_600_ measurements in accurately reflecting growth of sporulating strains, we analyzed the metabolic and biosynthetic capacity of sporulation-deficient strains using viable cell count (CFU/mL) as the biomass indicator at equivalent time points, as shown in Figure 6.

### 3.6. Effects of Sporulation Deficiency on Fluorescent Protein Expression

**Figure 7:**
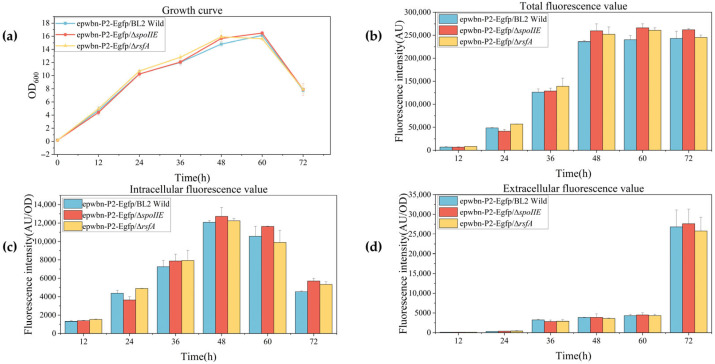
Fluorescence intensity detection of spore formation-deficient strains. (**a**) Growth curve of recombinant strains after 72 h of culture following the introduction of a fluorescent plasmid; (**b**) Total fluorescence intensity detection of recombinant strains after 72 h of culture; (**c**) Intracellular fluorescence intensity detection of recombinant strains after 72 h of culture; (**d**) Extracellular fluorescence intensity detection of recombinant strains after 72 h of culture.

Strains B1319 eGFP, Δ*spoIIE*-eGFP, and Δ*rsfA*-eGFP were activated on plates, and single colonies were inoculated into 15 mL LB medium supplemented with 7.5 μL kanamycin solution. After overnight cultivation for 16 h, cultures were inoculated at 3% into synthetic medium and cultivated at 37 °C with 220 rpm shaking for 72 h. Samples were collected for OD_600_ and fluorescence measurements. The experimental results are shown in Figure 7.

The sporulation-deficient strains carrying fluorescent expression plasmids showed no significant differences in growth compared to the control strain. During the early stages when spores had not formed or formed in limited numbers, fluorescence values were essentially identical to those of the control strain. Intracellular fluorescence peaked at 48 h, with values of 12,083, 12,731, and 12,253 for the control strain, Δ*spoIIE*, and Δ*rsfA*, respectively. Extracellular fluorescence reached maximum levels at 72 h, with values of 26,836, 27,626, and 25,787 for the control strain, Δ*spoIIE*, and Δ*rsfA*, respectively. After 48–72 h, total fluorescence values reached their peak and maintained relatively stable levels. Notably, sporulation-deficient strains consistently exhibited higher total fluorescence than the control strain, with the highest fluorescence intensity observed in the Δ*spoIIE* strain, which completely lacks sporulation capacity. Total fluorescence values were normalized against viable cell counts at corresponding time points.

**Figure 8:**
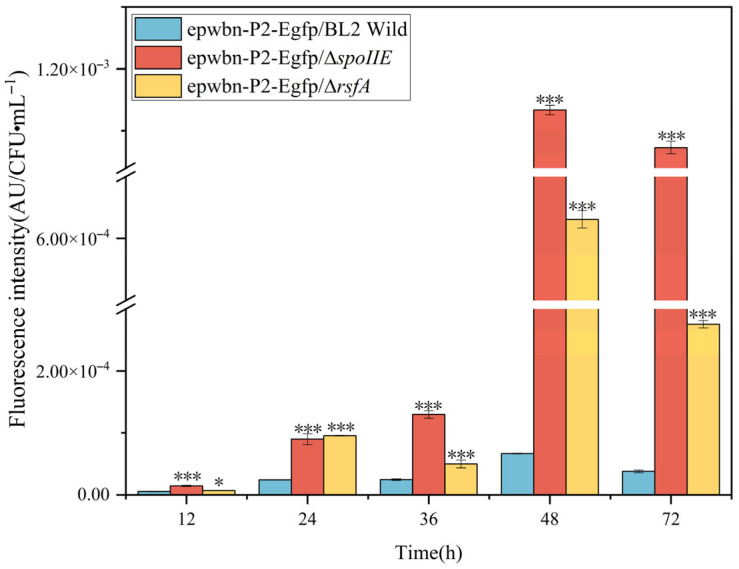
Fluorescence intensity detection of unit cells in sporulation-deficient strains. All experiments were independently repeated three times, and the average value was taken as the final result. The differences between two sets of data were analyzed using a 2-tailed Student’s *t*-test, while the differences between multiple sets of data were compared using one-way ANOVA and Tukey’s test. “*” and “***” were used to indicate the significance of *p* < 0.05 and *p* < 0.001, respectively.

**Figure 9:**
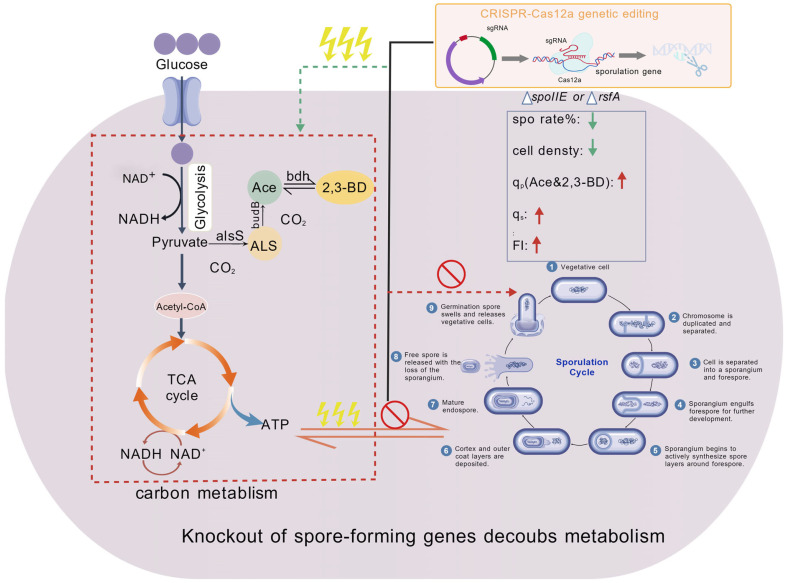
Schematic diagram of CRISPR-Cas12a-mediated knockout of sporulation genes and its metabolic decoupling effect. The up and down arrows represent increase or decrease, the bidirectional arrow represents that energy and matter can flow to each other through this pathway, energy is represented by a lightning symbol, if matter and energy cannot pass through this pathway, it is indicated by a prohibition symbol, a unidirectional horizontal arrow without a prohibition symbol indicates that matter and energy can be utilized through this pathway. This schematic illustrates the life cycle of spore-forming bacteria such as *Bacillus licheniformis* (1–8: sporulation stages; 9: germination stage) and the knockout of key sporulation genes using the CRISPR-Cas12a gene-editing system. By measuring parameters such as spore rate (%), cell density, production rates of acetoin and 2,3-butanediol (q_p_), substrate consumption rate (q_s_), and fluorescence intensity (FI), it demonstrates the decoupling effect between carbon metabolism (glycolysis, TCA cycle) and the sporulation development process following the knockout of sporulation genes.

As shown in Figure 8, the sporulation-deficient strain demonstrated significantly enhanced heterologous protein expression capacity per cell compared to the control strain. Figure 9 illustrates the major metabolic processes and potential energy flow involved in the gene knockout of the sporulating strain.

## 4. Discussion

This study successfully constructed sporulation-deficient strains of *B. licheniformis* and conducted comprehensive analyses of their growth and metabolic phenotypes. Contrary to expectations, blocking the sporulation pathway did not significantly alter substrate consumption or product synthesis curves at the culture level, but resulted in substantial improvements in per-cell metabolic capacity. These findings reveal the important regulatory role of sporulation processes in cellular growth and metabolism.

Sporulation is an Extremely “Expensive” Process. Under identical conditions, sporulation-deficient strains exhibited reduced or completely abolished sporulation rates, with viable cell counts substantially lower than wild-type strains. Research demonstrates that spore formation requires extensive consumption of peptidoglycan, proteins, and dipicolinic acid (DPA) [49], accompanied by complex gene expression regulation and morphological changes including asymmetric division [50], septum formation, engulfment, cortex synthesis, and coat assembly. These processes demand substantial ATP, NADPH, and NADP reducing power. Additionally, maintaining core dehydration requires energy input. This energy is primarily derived from glycolysis, the TCA cycle, and oxidative phosphorylation pathways [51]. Sporulation-deficient strains avoid this “wasteful” allocation of resources toward spore formation, redirecting more resources toward maintaining active vegetative cell metabolism and product synthesis.

Sporulation Deficiency Affects Cell Physiological Stability and Population Heterogeneity. Among strains with equivalent initial total biomass, sporulation-deficient strains exhibited several-fold reductions in viable cell counts, yet their overall metabolic activity and product synthesis did not decline. In fact, the metabolic capacity per unit cell increased several-fold. During growth and metabolism, strains consumed identical substrates and produced nearly equivalent quantities of products but relied on different numbers of effective working cells within their populations. In the late fermentation phase of wild-type strains, bacterial populations exist in highly heterogeneous states, simultaneously containing vegetative cells, cells at various stages of sporulation, released inactive mature spores, and senescent cells. These different cellular states exhibit vastly different metabolic activities. Vegetative cells serve as the primary working population during growth and metabolism of sporulating bacteria, while cells undergoing sporulation consume materials and energy but contribute minimally to product output [51]. Sporulation-deficient strain populations consist primarily of metabolically active vegetative cells, avoiding resource investment in sporulation by inefficient or counterproductive cells. This preserves vegetative cells with superior physiological states and higher efficiency, maintaining the cellular population in a more stable and uniform state, thereby enabling strains to focus more efficiently on target product synthesis.

Sporulation Deficiency Relieves Metabolic Regulatory Burden. Under identical conditions, sporulation-deficient strains exhibited increased fluorescent protein expression. Sporulation represents a massive regulatory network controlled by a series of cascading σ factors (*spo0A*, σ^F^, σ^E^, σ^G^, σ^K^), involving the specific expression and silencing of over 500 genes [10,11,12,13,14,15,16,17,18]. In wild-type strains, to ensure resource allocation for sporulation, cells shut down extensive gene expression related to vegetative growth, including basic metabolism, biosynthesis, and protein synthesis machinery. Even in the absence of sporulation-inducing stress conditions, maintaining this “shutdown” or “ready-to-shutdown” state itself requires energy and resource consumption, such as continuously expressed repressor proteins and non-coding RNAs [52]. In sporulation-deficient strains, sporulation initiation signals are blocked, and the global inhibitory capacity related to growth is greatly weakened. Fluorescent protein expression operates in an environment with fewer competing inhibitory factors, and host cells do not need to prepare for pathway shutdown. Consequently, the overall protein expression capacity of strains improves, and fluorescence intensity per unit cell increases significantly.

Competition and Balance Between Sporulation Deficiency and Cellular Metabolic Capacity. In sporulating bacteria, OD_600_ and viable cell counts exhibit asynchronous changes, making OD_600_ insufficient for representing the growth status of spore-forming bacteria. Viable cell counts provide a more accurate representation of bacterial biomass. Additionally, while impaired spore formation significantly reduces viable cell counts in strains, glucose utilization rates and production of 2,3-BD and acetoin show no significant differences. However, the specific glucose consumption rate and specific synthesis rates of 2,3-BD and acetoin per unit cell increase substantially. Studies demonstrate that OD_600_ measurements reflect the turbidity of total bacterial mass, including vegetative cells, spores, and dead cells, whereas viable cell counting (CFU) reflects reproductively capable living cells. During the stationary and decline phases of sporulating strains, culture turbidity decreases or remains constant due to spore formation and autolysis of vegetative cells, yet spore numbers may be high and stable at this time. Throughout the process from spore initiation to maximum sporulation rate, changes in OD_600_ and sporulation rate are not synchronous [43]. Therefore, viable cell counting is more suitable for assessing the growth status of spore-forming strains. Beyond the previously discussed benefits of sporulation deficiency—including resource conservation, regulatory burden relief, enhanced cellular stability, and simplified population heterogeneity—the improved substrate consumption and synthesis capacity per unit cell may also relate to altered energy and carbon flux in secondary pathways. Research has shown that when *Bacillus* species utilize glycolytic pathways to produce large amounts of pyruvate, substantial NADH generation occurs simultaneously. When excess NADH is produced, acetoin generated by microorganisms can utilize NADH to reduce itself to 2,3-butanediol, while NADH-dependent competitive byproducts such as lactate and ethanol also increase accordingly [53]. A competitive balance exists between sporulation and basal metabolism for energy and reducing power. On one hand, although partial glycolysis and TCA cycle activity occur during the early stages II and III of sporulation, cells tend to cease division and growth [8], resulting in decreased overall energy production efficiency. On the other hand, processes such as septum formation, engulfment, and spore structure synthesis require substantial energy and carbon skeletons [51]. This may lead to localized hypoxia and energy supply constraints, theoretically driving more glucose through secondary metabolic pathways like acetoin and 2,3 butanediol to balance NADH/NAD+. In contrast, the sporulation-deficient strains constructed in this study experience minimal or no interference from spore formation, enabling more efficient and uniform vegetative cells to continuously and stably perform basal metabolism for ATP generation while maintaining proper NADH/NAD+ balance, thereby optimizing energy and carbon flux. The efficiency of ATP generation through oxidative phosphorylation of NADH far exceeds that of acetoin and butanediol pathways, avoiding metabolic flux redirection or instability caused by energy and carbon source constraints due to sporulation morphogenesis and stress.

## 5. Conclusions

The CRISPR-Cpf1 knockout system was employed to achieve single-gene knockouts of the sporulation key genes *spoIIE* and *rsfA* in *Bacillus licheniformis*. Under optimized experimental conditions, a significant reduction in the sporulation rate was detected in the recombinant strains. Microscopic observation further revealed distinct morphological differences between the sporulation-deficient mutants and the parental strain. Sequencing analysis confirmed the successful deletion of the key sporulation genes *spoIIE* and *rsfA*. Subsequently, High-Performance Liquid Chromatography (HPLC) was used to assess the metabolite production of the sporulation-deficient strains. Additionally, the heterologous protein expression capability was evaluated using enhanced green fluorescent protein (eGFP) as a reporter gene. By analyzing the metabolic output and fluorescence intensity relative to the viable cell count (used as a substitute for OD_600_ measurement) at the same time point, it was determined that while the knockout of sporulation genes led to a decrease in overall biomass, the metabolic output per cell and heterologous protein expression were significantly enhanced. This process may involve a reallocation of resources and energy within the cell.

## Figures and Tables

**Table 1:** List of all strains and plasmids used or constructed in this study.

Strain or Plasmid	Features or Uses	Source
*Escherichia coli* JM109	abbreviated as JM109, a molecular cloning host	laboratory preservation
*Bacillus licheniformis* CICIM B1319	abbreviated as B1319, wild-type strain	laboratory preservation
JMpE	JM109 carrying plasmid pE	this study constructs
JMpA	JM109 carrying plasmid pA	this study constructs
BLpE	B1319 carrying plasmid pE	this study constructs
BLpA	B1319 carrying plasmid pA	this study constructs
Δ*spoIIE*	recombinant strain after BLpE knockout of the *spoIIE* gene resulting in the loss of plasmid pE	this study constructs
Δ*rsfA*	recombinant strain after BLpA knockout of the *rsfA* gene resulting in the loss of plasmid pA	this study constructs
B1319-Egfp	B1319 strain carrying the epWBN-P2-eGFP plasmid	this study constructs
Δ*spoIIE*-eGFP	Δ*spoIIE* strain carrying the epWBN-P2-eGFP plasmid	this study constructs
Δ*rsfA*-eGFP	Δ*rsfA* strain carrying the epWBN-P2-eGFP plasmid	this study constructs
pJOE8999-Ppro-cas12a-*spoIIE*	pJOE8999 expression vector, containing crRNA targeting *spoIIE* and homologous arm sequences, referred to as pE	this study constructs
pJOE8999-Ppro-cas12a-*rsfA*	pJOE8999 expression vector, containing crRNA targeting *rsfA* and homologous arm sequences, referred to as pA	this study constructs

**Table 2:** The reagents and kits used in this study.

Product	Production Company
restriction endonuclease	Thermo Fisher Scientific, USA (168 Third Avenue, Waltham, MA, USA)
2 × Taq/Phanta PCR Master Mix	Nanjing Novizan Biotechnology Co., Ltd. (Nanjing, Jiangsu, China)
plasmid DNA extraction kit, DNA purification kit	Nanjing Novizan Biotechnology Co., Ltd.
DNA Molecular Weight Standard Marker	Takara Bio Co., Ltd. (Nojihigashi 7-4-38, Kusatsu, Shiga, Japan)
Kanamycin	Merck Sigma Company (St. Louis, MO, USA.)
peptone, yeast extract, agar powder	OXOID Company, UK (Basingstoke, England, UK)

**Table 3:** Instruments used in this study.

Instrument	Manufacturer
constant temperature metal bath	Shanghai Yiheng Technology Co., Ltd. (Shanghai, China)
fully automatic high-pressure steam sterilizer	Sanyo Corporation Japan (Osaka, Japan)
S100D PCR Machine	BIO-RAD Company, USA (Hercules, CA, USA)
DYY-6C Nucleic Acid Electrophoresis Apparatus	Beijing No. 61 Factory (Beijing, China)
Chemi Doc XRS Gel Imaging System	BIO-RAD Company, USA
PICO17 High-Speed Centrifuge	Thermo Company, USA (Waltham, MA, USA)
Ultraviolet Spectrophotometer (UV-1200)	Shanghai Meipuda Instrument Co., Ltd. (Shanghai, China)
cleanroom Workbench	Dalian Baosheng Bioengineering Co., Ltd. (Dalian, Liaoning, China)
SPARK Multi-Mode Microplate Reader	Shanghai Dicon Trading Co., Ltd. (Shanghai, China)
ultrasonic cleaner	Ningbo Xinzhi Biotechnology Co., Ltd. (Ningbo, Zhejiang, China)
ultra-low temperature freezer	Japan HITACHI Company (Tokyo, Japan)
XSP-13C-LP phase-contrast microscope	Shanghai Procision instruments Co., Ltd. (Shanghai, China)

**Table 4:** The primers used in this study to construct the plasmids.

Primer Name	Primer Sequence (5′-3′)
*spoIIE*-crRNA-F	aaagcaatgagacgatcaagctgAATTTCTACTGTTGTAGATCAAATAAAACG
*spoIIE*-crRNA-R	AATTcagcttgatcgtctcattgctttATCTACAACAGTAGAAATTAAATGCTCC
*spoIIE*-Left-F	CTGAAAAGTTTATACCCGGGcttatcccaacggatgcctt
*spoIIE*-Left-R	tctttccgctttttccatgc
*spoIIE*-Right-F	gcatggaaaaagcggaaagaagaatcaagagatttcttagccttc
*spoIIE*-Right-R	TTTTTACCCGGTACCTGGATCCacatgaagatgtacggtctcg
*rsfA*-crRNA-F	ctgcttgctgaaacggtattgcgAATTTCTACTGTTGTAGATCAAATAAAACG
*rsfA*-crRNA-R	AATTcgcaataccgtttcagcaagcagATCTACAACAGTAGAAATTAAATGCTCC
*rsfA*-Left-F	CTGAAAAGTTTATACCCGGGcaaaaacgcctgacccgtta
*rsfA*-Left-R	ccaagcgtcttgtctttgtttc
*rsfA*-Right-F	atgaaacaaagacaagacgcttggggcaacttagaaaaaatggctg
*rsfA*-Right-R	TTTTACCCGGTACCTGGATCCttttgatgagaagataagccgcc
pJOE8999-F	TATCTACAACCATCACTGTACCTC
pJOE8999-R	CGTAACAGCAAAACAGGTACTGAA
Δ*spoIIE*-YZ-F	agaggttaacttgctgctttcta
Δ*spoIIE*-YZ-R	ttgagactgatggaaaggtctat
Δ*rsfA*-YZ-F	ctttttttcaaacgtgaggcaaac
Δ*rsfA*-YZ-R	ttggatagaagaagagcctctg
epWBN-eGFP-F	AGCCCAAAAATAATCCAACAATTCT
epWBN-eGFP-R	TGCTGAAGCTAGCTTGCATG

**Table 5:** The target sequences designed in this study.

Name	Combined Sequence
crRNA-*spoIIE*	aaagcaatgagacgatcaagctg
crRNA-*rsfA*	ctgcttgctgaaacggtattgcg

**Table 6:** Expression and regulation of sporulation-related genes at different stages.

Sporulation Stage or Regulon	Sporulation Gene(s)
	Conserved Mostly in *Bacillus*
Conserved mostly in *Bacillus*	*spo0A*, *sigH*, *spoIIE*, *spoIIIE*, *spoIIIJ*, *pth*, *spoVG*, *spoVS*, *divIB*, *divIC*, *divIVA*, *ftsA*, *ftsE*, *ftsH*, *ftsX*, *ftsY*, *ftsZ*, *jag*, *minC*, *minD*, *minJ*, *obgE*, *sweC*, *spo0B*, *spo0F*, *spo0E*, *ald*, *ftsL*, *ymcA*, *ylbF*, *yaaT*, *sda*
Spo0A regulon	*sigE*, *sigF*, *sigG*, *spoIIAA*, *spoIIAB*, *spoIIGA*, *parA*, *parB*, *yisK*, *yusE*
Engulfment	*spoIID*, *spoIIM*, *spoIIP*, *spoIIQ*, *spoIIIAA*, *spoIIIAB*, *spoIIIAC*, *spoIIIAD*, *spoIIIAE*, *spoIIIAF*, *spoIIIAG*, *spoIIIAH*, *spoIIB*, *spoIIIL*, *yunB*
Forespore-expressed genes SigF regulon	*spoIIR*, *spoIVB*, *spoVT*, *dacB/dacF*, *ytfJ*, *ydfS/yetF*, *yhcV/ylbB*, *ylbC*, *yloC*, *yuiC*, *yyaC*, *bofC*, *rsfA*, *yabK*, *yjbA*, *ymfJ*, *yqhG*, *ywzB*
SigG regulon	*spoVAC*, *spoVAD*, *spoVAEB*, *yqfS*, *sspA/sspB/sspC/sspD*, *spoVAA*, *spoVAB*, *spoVAF*, *sspE*, *sspF*, *sspH*, *sspI*, *tlp*, *yqfX*
Mother cell-expressed genes SigE regulon	*sigK*, *spoIIID*, *spoIVFB*, *spoVB/ykvU*, *spoVE*, *spoVK*, *ctpB*, *dacB/dacF*, *yncD*, *spmA*, *spmB*, *yisY/yfhM*, *yitE/yqfU*, *ylmC/ymxH*, *ytaF*, *ytvI*, *yyaD/ykvI*, *spoIVFA*, *spoVM*, *bofA*, *ydcA*, *ydcC*, *yhbH*
SigK regulon	*spoVFA*, *spoVFB*, *cgeD*, *ykuD/yciB*, *ytdA*, *ytlD*
Spore cortex	*spoVD*, *ylbJ*, *cwlC/cwlD*, *lytH*, *spoIVH*, *yabP*, *yabQ*, *yqfC*, *yqfD*, *cotD*
Spore coat	*spoIVA*, *cotJC/yjqC*, *cotSA*, *gerM*, *ycsK*, *safA*, *sleL/ydhD*, *spoVID*, *spoVIF*, *cotE*, *cotJA*, *cotJB*, *cotM/cotP*, *yhjR*
Germination	*gerA*, *gerB*, *gerC*, *lgt*, *gpr*, *cwlJ/sleB*, *cspA*, *gdh*, *gerD*, *gerE*, *gerQ*, *ypeB*

## Data Availability

The original contributions presented in this study are included in the article. Further inquiries can be directed to the corresponding author.

## Abbreviations

The following abbreviations are used in this manuscript:

2,3-BD	2,3-butanediol
DPA	dipicolinic acid
Pyl	pyruvate
Ace	acetoin
ALS	acetolactate
FI	fluorescence intensity
